# Effects of familial risk and stimulant drug use on the anticipation of monetary reward: an fMRI study

**DOI:** 10.1038/s41398-019-0399-4

**Published:** 2019-02-04

**Authors:** Alanna L. Just, Chun Meng, Dana G. Smith, Edward T. Bullmore, Trevor W. Robbins, Karen D. Ersche

**Affiliations:** 1 Departments of Psychiatry and Psychology, University of Cambridge, Cambridge, UK; 2 Behavioural and Clinical Neuroscience Institute, University of Cambridge, Cambridge, UK; 3 Cambridgeshire and Peterborough NHS Foundation Trust, Cambridge, UK; 4 GlaxoSmithKline, Immuno-Inflammation Therapeutic Area Unit, Stevenage, UK

## Abstract

The association between stimulant drug use and aberrant reward processing is well-documented in the literature, but the nature of these abnormalities remains elusive. The present study aims to disentangle the separate and interacting effects of stimulant drug use and pre-existing familial risk on abnormal reward processing associated with stimulant drug addiction. We used the Monetary Incentive Delay task, a well-validated measure of reward processing, during fMRI scanning in four distinct groups: individuals with familial risk who were either stimulant drug-dependent (*N* = 41) or had never used stimulant drugs (*N* = 46); and individuals without familial risk who were either using stimulant drugs (*N* = 25) or not (*N* = 48). We first examined task-related whole-brain activation followed by a psychophysiological interaction analysis to further explore brain functional connectivity. For analyses, we used a univariate model with two fixed factors (familial risk and stimulant drug use). Our results showed increased task-related activation in the putamen and motor cortex of stimulant-using participants. We also found altered task-related functional connectivity between the putamen and frontal regions in participants with a familial risk (irrespective of whether they were using stimulant drugs or not). Additionally, we identified an interaction between stimulant drug use and familial risk in task-related functional connectivity between the putamen and motor-related cortical regions in potentially at-risk individuals. Our findings suggest that abnormal task-related activation in motor brain systems is associated with regular stimulant drug use, whereas abnormal task-related functional connectivity in frontostriatal brain systems, in individuals with familial risk, may indicate pre-existing neural vulnerability for developing addiction.

## Introduction

A change in our understanding of drug addiction as a brain disorder was influenced by the notion that drug addiction was not a deficit of character, but rather the result of aberrant brain function caused by excessive drug use^1^. A prominent hypothesis has been that drugs of abuse alter reward processing through disruption of the mesolimbic dopamine reward system – a theory that has since been supported by both animal models and human research^2^. The brain reward system assimilates both top-down and bottom-up inputs from cortical and subcortical brain circuitry, including prefrontal and motor cortical systems, to support the integrated evaluation of environmental stimuli^3^. Dysregulation of the mesolimbic dopamine reward system has been associated with both acute and chronic use of stimulant drugs^4,5^, leading to changes in how drug users perceive and process reward. These changes hypothetically facilitate the development and maintenance of addiction^6,7^.

However, the notion that drug use is the sole cause of aberrant reward processing in addicted individuals is disputable. Evidence indicates that stimulant drug users with a family history of substance abuse are eight times more likely to develop an addiction than those without a family history^8^. Familial transmission of substance use disorders suggests pre-existing genetic^9,10^, sociological, economic, and other environmental risk factors that are often shared between biological first-degree relatives (i.e., familial risk-factors). Accordingly, healthy first-degree relatives of individuals affected by addiction report a number of abnormalities in terms of personality traits and brain structure^11,12^, aberrant striatal dopamine neurotransmission^13–15^, and altered striatal function during reward processing^16,17^. Thus, pre-existing familial vulnerability renders the individual vulnerable to developing addiction should they start taking drugs^18–23^. Likewise, stimulant drugs also exert effects that contribute to aberrant reward processing and problematic drug use^24,25^.

The potential contribution of both familial and drug use-related risk factors makes it difficult to determine the cause of aberrant reward processing associated with stimulant drug use. Here, we investigated the effects of stimulant drug use and familial risk within a single statistical model to determine their possible separate and interacting effects on reward processing. To identify the effects of familial risk we examined candidate endophenotypes based on neural network activation patterns. *Endophenotypes* are stable, heritable, and quantifiable traits observed in both clinically affected individuals and their unaffected first-degree relatives^26^. Hypothetically, endophenotypic abnormalities, arising from genetic, sociological, economic, and other environmental risk-factors common between biological first-degree relatives (i.e., familial risk-factors), both subserve and predate the development of stimulant drug addiction.

To identify endophenotypes in the current study, we included fully-related siblings who not only share approximately 50% of their genes, but also childhood sociological, economic, and other familial risk-factors, which may influence the development of addiction^27,28^. These individuals are of particular interest not only because of their increased familial risk of developing addiction but also because of their success in avoiding the initiation of stimulant drug use despite the increased risk, potentially indicating compensatory or resiliency mechanisms. This endophenotype approach has been used previously in addicted populations^11,12,29,30^, although not in relation to reward processing. To identify effects of stimulant drug use as well as the familial risk of addiction within the same model, we included non-dependent stimulant drug users and non-drug users, both without familial risk, in addition to the sibling pairs with familial risk. Together, the four distinct groups each possessed a unique combination of familial and stimulant drug-related risk-factors, allowing the disentanglement of the distinct and interacting effects of both stimulant drug use and familial risk. By investigating interacting effects, we may gain insight into possible compensation or resiliency in at-risk individuals, such as those with familial risk who successfully avoided stimulant drug use, and those who use stimulant drugs but successfully avoid the development of addiction.

Although the risk associated with the development of addiction may not be drug-specific, we deliberately focused on stimulant drugs given their critical dependence on the mesocorticolimbic dopamine system^31,32^ and the hypothesized association between the drug-induced release of dopamine and the drug’s addictive liability^33^. The abuse liability of stimulant drugs and the relative high heritability, which has been estimated to be 0.72^33^, suggest that individual variations in behaviorally relevant neural networks may mediate the individual’s susceptibility to drug addiction^33^.

We examined neural responses to reward using the monetary incentive delay (MID) task—a well-validated paradigm for examining anticipatory brain responses to reward^34,35^. The MID-task has been associated with abnormal activation in frontostriatal circuits in drug users and those at-risk for addiction; however, these results have been inconsistent^25^ and often focus on regions of interest rather than the whole brain. More importantly, prior studies have not addressed the critical question of causality. To address this question in stimulant drug addiction, we hypothesized that if aberrant reward processing is a consequence of stimulant drug use, then participants with a personal history of stimulant drug use will show altered task-related striatal function during reward processing. Alternatively, if aberrant reward processing is a pre-existing vulnerability resulting from familial risk, then fully-related sibling pairs will share a similar pattern of abnormal task-related striatal function during reward processing distinct from that of unrelated control participants.

## Materials and methods

### Study sample

A total of 165 participants were recruited for this study from local treatment centers, by media advertisements, or by word-of-mouth within the community. All participants were between the age of 18–55 years and fluent in written and spoken English. Participants also underwent semi-structured interviews to ascertain personal and family history of drug/alcohol addiction, physical health (including signs of acute intoxication and withdrawal), and mental health as assessed with the Structured Clinical Interview for DSM-IV-TR Axis I Disorders^36^. Participants were split into four groups based on their individual familial risk (F) and stimulant drug-related risk (S). Stimulant-dependent individuals (F+S+) and their unaffected siblings (F+S−) composed the groups with familial risk. Non-dependent stimulant drug users without a family history of addiction (F−S+) and individuals without either family or personal history of drug addiction (F−S−) composed the control groups without familial risk (see Table 1).

**Table 1:** Demographics, personality, clinical and MID-task performance data for all participants

	F–S− No familial risk; No stimulant use (*N* = 48)		F−S+ No familial risk; Stimulant use (*N* = 25)		F+S− Familial risk; No stimulant use (*N* = 46)		F+S+ Familial risk; Stimulant use (*N* = 41)	
	Mean	(±SD)	Mean	(±SD)	Mean	(±SD)	Mean	(±SD)
*Demographics*								
Age (years)	32.5	( ± 8.8)	28.6	(±6.6)	32.3	(±8.4)	34.6	(±7.4)
Gender (% male)	63%		52%		48%		90%	
Disposable income (£/month)	660	(±940)	714	(±1154)	403	(±411)	399	(±672)
Trait Impulsivity (BIS-11 total score)	59.3	(±7.6)	63.2	(±10.4)	67.2	(±10.4)	77.0	(±9.4)
Alcohol consumption (AUDIT total score)	3.2	(±2.3)	5.8	(±1.5)	3.9	(±4.6)	11.7	(±11.9)
Drug-taking experiences (DAST-20 total score)	0.0	(±0.0)	2.4	(±1.0)	0.5	(±1.1)	Not completed	
Compulsive use of stimulants (OCDUS total score)	-	-	1.2	(±1.7)	-	-	23.6	(±9.3)
Nicotine Use (current/past)	12.5%	43.8%	68%	16%	54%	37%	92.7%	4.9%
Cannabis Use (current/Past)	0%	20.8%	36%	60%	8.7%	65.2%	65.9%	34.1%
*Task performance*								
Money gained (£)	8.65	(±1.2)	8.46	(±1.2)	8.48	(±1.2)	8.49	(±1.5)
Successful responses to neutral trials (number)	12.4	(±2.1)	12.1	(±1.8)	12.1	(±2.4)	10.2	(±3.1)
Successful responses t rewarding trials (number)	27.9	(±3.4)	28.6	(±2.9)	27.8	(±3.6)	27.9	(±4.9)
Response time for successful neutral trials (ms)	210.6	(±23.7)	206.7	(±22.4)	211.5	(±23.1)	220.6	(±40.1)
Response time for successful rewarding trials (ms)	204.5	(±19.0)	200.8	(±19.7)	208.3	(±21.2)	207.0	(±25.8)

Data are displayed by individual group status. Standard deviation (SD) shown in parentheses. [Notes: AUDIT score > 8 indicate harmful drinking. DAST-20 < 5 indicate recreational use of drugs in general (not specific to stimulant drugs). The DAST-20 test was not administered in dependent stimulant users as it is not sensitive to clinical populations

All F+S+ participants were required to satisfy the DSM-IV-TR criteria^37^ for stimulant drug dependence (cocaine: 92.7%; amphetamines: 7.3%) and share both biological parents with an F+S− sibling who was also able to participate in the study. Additionally, F−S+ control participants were required to have engaged in regular stimulant drug use for at least two years but had never developed addiction to drugs or alcohol and had never been prescribed stimulant drugs for medical reasons. We intentionally recruited *non-dependent* stimulant drug-using controls because their lack of dependence reduced their likelihood of possessing those familial risk factors and associated neural vulnerabilities that render individuals susceptible to addiction. The sample size was determined by power analysis, establishing a group size of *N* = 42 for 95% power to identify the effects of familiarity. Semi-structured interviews determined that control participants had no first-degree relative affected by addiction.

For all groups, exclusionary criteria included a lifetime history of a psychotic, neurological, or neurodevelopment disorder, or traumatic head injury. Exclusion criteria for unaffected siblings and unrelated controls also included any personal history of substance addiction (except nicotine). Concurrent drug and alcohol consumption for these three groups were low (as reflected by the Alcohol Use Disorders Identification Test [AUDIT] and Drug Abuse Screening Test [DAST-20] scores, see Table 1). Experience with tobacco and nicotine have been reported in all groups (see Table 1). Group differences in reported nicotine (χ^2^ = 67.04, *p* < 0.001) and cannabis use (χ^2^ = 104.36, *p* < 0.001) between stimulant drug users and non-users were controlled for in a separate post-hoc analysis. Critically, although drug-taking experiences are common in people with familial risk^38^, F+S− participants in the present study did not report stimulant drug use. Exclusion criteria were kept deliberately minimal as minor psychopathology may be a clinical marker of vulnerability and an important characteristic of the participant in the familial risk group. Further details can be found in the supplementary material (SM).

In addition to stimulant drug dependence, 12 F+S+ participants further met diagnostic criteria for alcohol dependence, and 22 F+S+ participants for opioid dependence. Consistent with prior literature^39,40^, participants with familial risk reported a high prevalence of childhood adversity when compared with control participants (F_1,159_ = 29.12, *p* < 0.001), as determined by Childhood Trauma Questionnaire (CTQ) abuse scores^11,41^. Differences in familial relationship between sibling pairs and unrelated control groups (i.e., sibling pairs with familial risk were related, whereas control participants without familial risk were not) was investigated post-hoc. Prior to testing, stimulant-positive urine samples were provided by all except three F+S+ participants, indicating the use of stimulant drugs in the last 72 h^42^. Drug-negative urine samples were provided by all other participants. This study was approved by the NHS Cambridgeshire2 Research Ethics Committee (08/H0308/310PI:KDE), and written informed consent was obtained from all participants prior to study enrollment. This sample is described in more detail in the SM; separate data from this sample have been published previously^11,12,43–48^.

### MID-task design

**Fig. 1:**
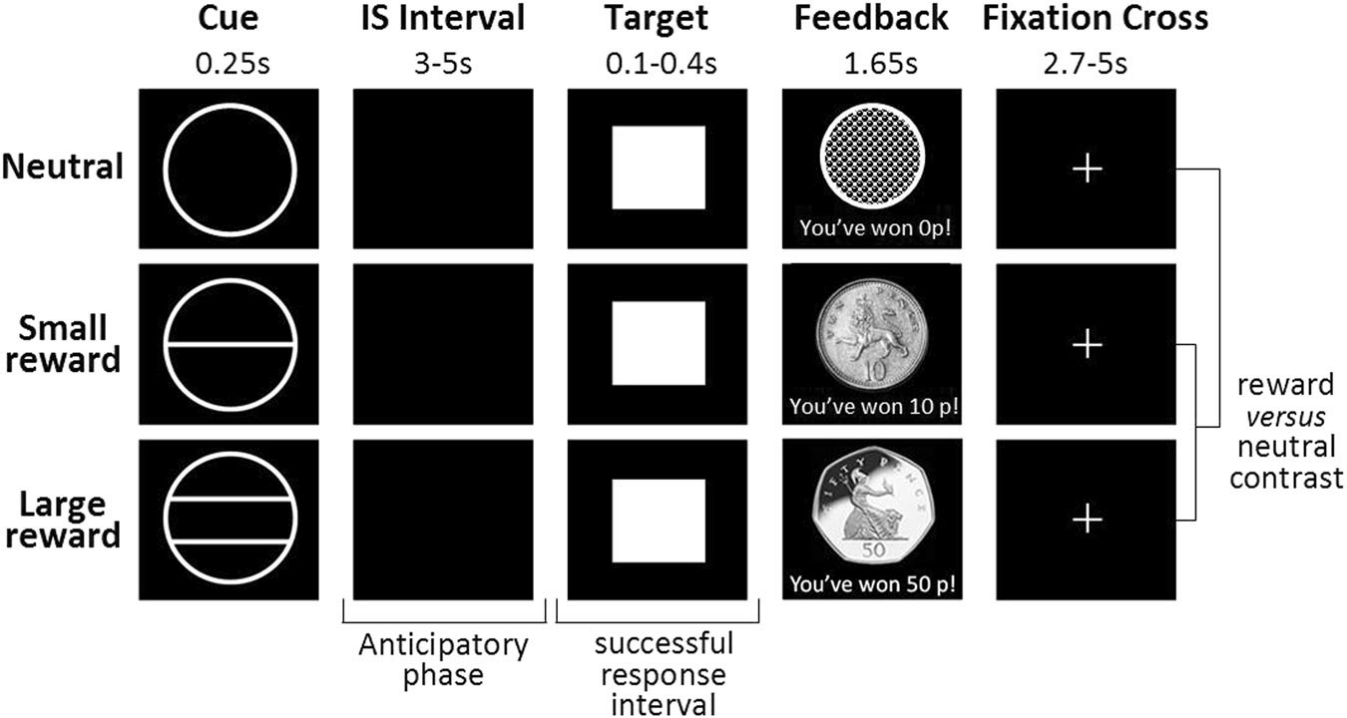
Schematic representation of the MID-paradigm depicting screen sequence, contrast used, and key performance variable intervals. The sequence presented screens including an anticipatory cue, inter-stimulus (IS) interval, target stimulus, feedback, and fixation cross. The neutral cue (no reward) was represented by an empty circle, the small gain (10 pence) cue was represented by a circle transected by a single horizontal line, and the large gain (50 pence) cue was represented by a circle transected by two horizontal lines. Successful feedback was depicted by an image of a 10p or 50p coin with the words “you’ve won 10p!” and “you’ve won 50p!” respectively (depending on the magnitude of the previous anticipation cue). Unsuccessful feedback (too late or too early) and successful neutral reward feedback, was depicted as an empty circle with the words “you’ve won 0p!”

We used the MID-task^49^ to examine the neural correlates of reward anticipation (Fig. 1). The task consisted of three phases: anticipation, target, and feedback. A cue presented in the anticipation phase notified participants of the potential to receive a monetary or neutral reward. The target stimulus, presented following the anticipation phase, prompted participants to respond by pressing a button. Finally, during the feedback phase participants were informed about the outcome of their behavioral response.

During the anticipatory phase, three cues, indicating either a neutral or rewarding outcome, were randomly displayed (Fig. 1). The anticipatory cue was presented for 250 ms, notified participants of the potential to win a monetary or a neutral reward, and was followed by a jittered black anticipation screen for 3000–5000 ms. The target stimulus, always a white square, was then presented for 100–400 ms, during which the participant had to respond by pressing a button. Immediately following the target stimulus, either successful or unsuccessful feedback, contingent on participant response, was presented for 1650 ms. Feedback screens were followed by a fixation cross screen, which was presented for 5000–2700 ms until the next trial commenced. Responses were deemed successful if performed during presentation of the target on the screen. The time between the presentation of the cue and the target stimulus represents the anticipatory phase^50^, during which neural responses were recorded (Fig. 1).

Prior to scanning, all participants underwent 66 training trials to ensure they had a clear understanding of how to complete the task. Participants were told prior to scanning that their total earnings were contingent upon task performance. The duration of the anticipation phase was based on participants’ performance to maintain a 66% success rate. In total, 66 trials were completed by each participant. Participants also completed an additional, but separate version of this task, which included drug-related cues, the results of which are not reported here.

### Statistical analysis

Behavioral data were analyzed using the Statistical Package for Social Science (SPSS v.22; IBM Chicago, Illinois). We employed a univariate analysis of variance model with two fixed between-subject factors: *familial risk* (participants with and without addiction in the family) and *stimulants* (participants with and without stimulant drug use). This statistical model allowed the separate investigation of familial risk and stimulant drug use as well as possible interactions between these two factors. Analyzing distinct and interacting effects within the same model not only mitigates Type I error but can also reveal interactions between these two factors. This is particularly important as the development of addiction is likely contingent on both the use of drugs and familial risk-factors^28,51^.

Gender and monthly disposable income were included as covariates in all analyses due to their reported involvement in reward processing and the development of addiction^52,53^, and to control for group differences (gender: χ^2^ = 7.01, *p* = 0.008, income: F_1,155_ = 5.40, *p* = 0.021). These differences were driven by male dominance in the F+S+ group and less monthly disposable income in participants with familial risk, irrespective of drug use. Participants with less money to spend demonstrated decreased response times (*r* = −0.18, *p* = 0.022). Given that age did not differ between groups (F_3,155_ = 2.60, *p* = 0.054) and was not correlated with outcome measures, it was not used as a covariate.

We controlled, post-hoc, for the potentially confounding differences in the severity/duration of drug use between the two stimulant using groups, and the different relationships between participants in the groups with and without familial risk. Accordingly, we subjected the significant neuroimaging results to a one-way ANCOVA analysis by importing mean cluster beta values (i.e., mean activation or connectivity values) into SPSS. To control for differences in drug-taking experiences, we entered the following variables as covariates: severity of stimulant use (OCDUS score), years of stimulant drug use, alcohol consumption (AUDIT score), current/past nicotine use, and current/past cannabis use. To control for the shared environment of the sibling pairs, we further included a measure of childhood adversity (CTQ abuse score) as a covariate in the same post-hoc ANCOVA model. We did not include these variables as covariates in the main analysis to avoid statistically controlling for aspects that were critical in defining the groups^54^.

Behavioral data were analyzed for accuracy and latency of successful trials, and the total amount of money gained using a repeated-measures ANCOVA model with trial type (monetary versus neutral reward) as the within-subject factor and *stimulants* and *familial risk* as the two between-subject factors. Square-root transformation was used for best normalization. Sidak post-hoc analysis was used for all individual group comparisons. Accordingly, demographic data were analyzed using a general linear model (GLM) with *stimulants* and *familial risk* as fixed factors. Demographic and task performance data were used for analyses (see Table 1). Pearson correlation coefficients were calculated to assess the relationships between outcome measures. We also examined the relationship between self-reported impulsivity and task-related neural activation and functional connectivity using the Barratt Impulsiveness Scale (BIS-11). All tests were two-tailed and significance levels of *p* < 0.05 were assumed.

### Neuroimaging data acquisition and analysis

Further details on neuroimaging data acquisition and analysis can be found in the SM. Briefly, neuroimaging data were acquired at the Wolfson Brain Imaging Centre, University of Cambridge, in one run on a Siemens TIM-Trio 3-Tesla scanner (Siemens Medical Solutions, Erlangen, Germany). The scans were analyzed in FMRIB’s Software Library (FSL, v-5.0.9, https://fsl.fmrib.ox.ac.uk/fsl) and consisted of two main stages: (1) task activation analysis to identify brain regions involved in reward processing and effects of interest (i.e., main effects of familial risk, stimulant drug use, and their interaction); (2) post-hoc psychophysiological interaction (PPI) analysis to further explore functional connectivity related to reward anticipation and effects of interest.

Following standard pre-processing procedures, statistical analyses were conducted at the first and second levels by using fMRI Expert Analysis Tool. At the first level, GLM analysis was conducted for each participant. As reward magnitude showed no behavioral differences in latency, accuracy, or brain activation, small and large rewards were collapsed resulting in a single monetary reward type. Contrasts of interest included: anticipation of monetary reward versus neutral reward, successful feedback, and unsuccessful feedback (results on feedback are reported in the SM), consistent with prior literature^34,35^.

At the second level, the main effect of each contrast collapsed across groups was computed using one-sample *t*-tests, with gender and monthly disposable income as covariates, to reveal task activation patterns. The ANCOVA model was used to assess main effects of *stimulants* and *familial risk* in the four groups as well as their interaction for each contrast. In the second stage, owing to observed task-related activation in the putamen, post-hoc exploratory PPI analysis was used between striatal seed regions of interests (ROI) and whole brain. The seed ROI was defined by the peak coordinates of the significant putamen cluster extracted from a group level with a 4 mm radius. For all analyses, significant effects were defined, after voxelwise testing and family-wise error (FWE) correction for multiple comparison, as clusters with a voxel height of Z > 2.58 (i.e., *p* < 0.005) and cluster-corrected *p*-value of 0.00015 (using FSL’s easythresh function). Due to recent discussions regarding the control of false positives in cluster inference^55^, we employed a relatively more stringent threshold than the conventional Z > 2.3 (i.e., *p* < .01).

Of the nine participants that were excluded post-hoc, five were due to excessive head motion during scanning (1 F−S−, 3 F+S+, 1 F+S−), one due to poor data quality (1 F+S−), and three due to incomplete behavioral data (1 F+S +, 1 F+S−, 1 F−S+), resulting in a remaining sample of 160 participants. Baseline demographic characteristics did not differ between excluded and included participants.

## Results

### Task performance

**Fig. 2:**
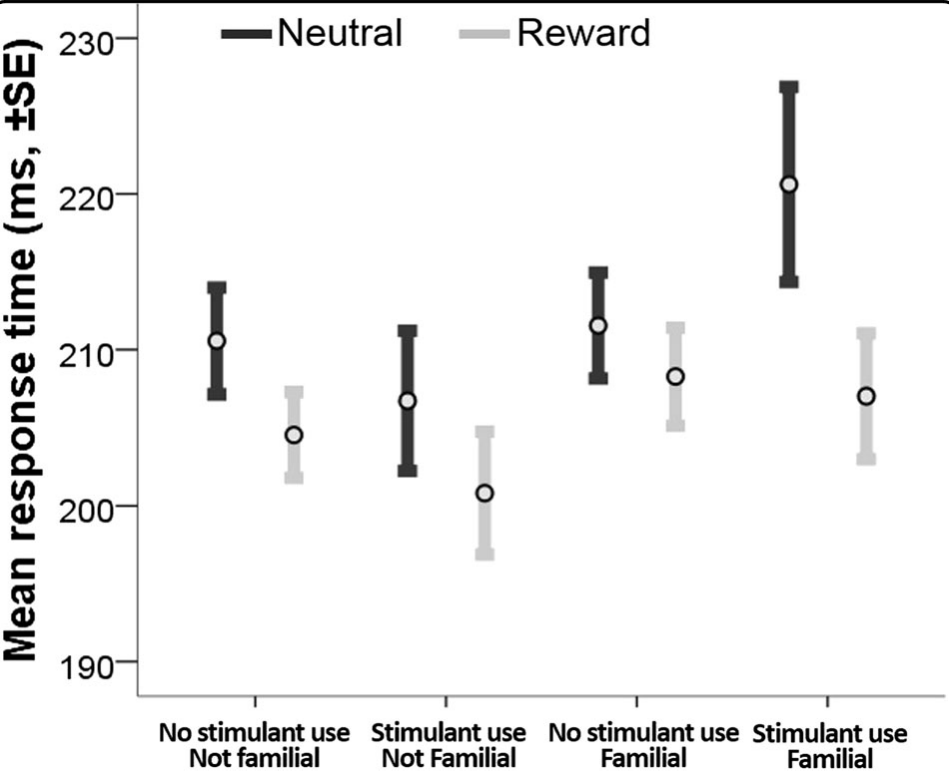
Mean response latency in anticipation of monetary and neutral rewards. All participantes responded faster in anticipation of monetary reward compared to neutral reward

In keeping with the MID-task design to minimize behavioral differences, we did not find a main effect of *familial risk* or *stimulants* on performance measures (Table 1). However, since the MID-task included both monetary and neutral reward trials, we analyzed, post-hoc, the effect of trial valence on accuracy and latency of successful trials. In accordance with prior work, we observed across all groups, a main effect of trial valence on the number of successful responses (F_1,155_ = 218.55, *p* < 0.001), and mean response times (F_1,155_ = 22.23, *p* < 0.001; Fig. 2), such that participants responded faster and with more accuracy to the prospect of a monetary reward compared with a neutral reward. Detailed performance data are summarized in Table 1.

### Task-related brain activation

**Fig. 3:**
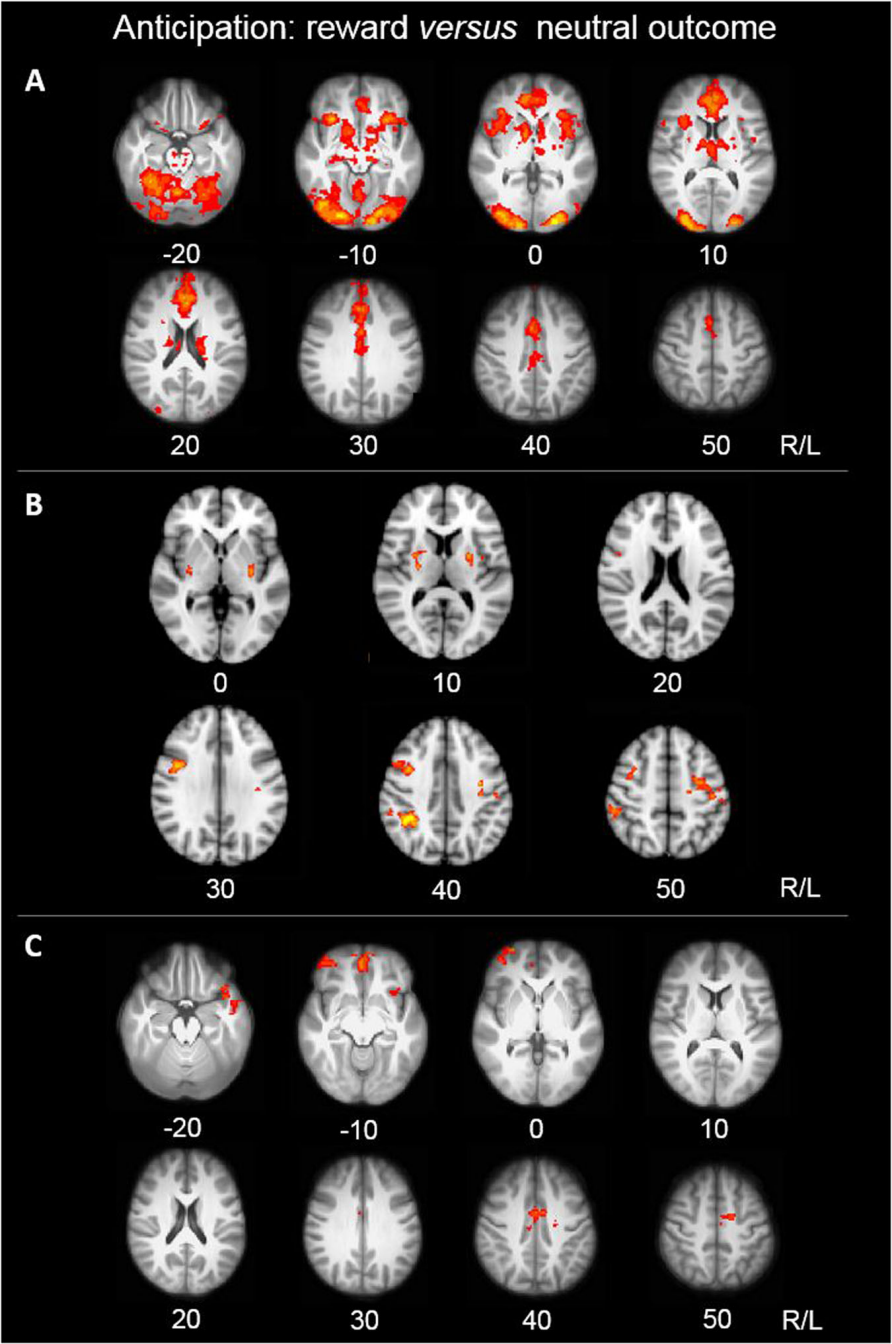
Brain function during the anticipation of monetary reward *versus* neutral reward. Yellow depicts relatively greater changes in activation or functional connectivity. Z-coordinates represented in MNI space. **a** MID-task activation across all groups. Thresholded statistical map shown (*p* < 0.00015; FWE corrected for multiple comparisons). **b** Effect of stimulant drug use on task-related brain activation. Thresholded statistical map showing areas of increased activation in stimulant drug users compared with non-users (*p* < 0.00015; FWE corrected for multiple comparisons). **c** Effect of familial risk on putamen functional connectivity to the whole brain. Thresholded statistical map showing areas of altered functional connectivity in familial groups compared with non-familial groups (*p* < 0.00015; FWE corrected for multiple comparisons)

All significant clusters resulting from the whole brain neuroimaging analysis are reported in Table 2. Results describe brain function during reward anticipation and are reported at cluster-corrected levels using the aforementioned thresholds (see Methods for details). Consistent with prior work^25^, the MID-task activated reward-related regions in all participants. Significant clusters of task-related activation encompassed the ventromedial orbitofrontal cortex (OFC) and occipital pole, extending to the paracingulate gyrus, anterior cingulate cortex (ACC), insula, pallidum, striatum, and thalamus (Fig. 3a; Table 2).

**Table 2:** Peak *Z*-values and MNI-coordinates.

Brain region	BA	Peak Z-value	Peak coordinates		
			*x*	*y*	*z*
*A. Task-related activation*					
*Anticipation condition*					
*Task activation*					
Orbital frontal cortex*	13	5.98	30	22	−10
Occipital pole	18	7.41	−22	−96	4
*Stimulant use* > *no stimulant use*					
Right dorsolateral putamen	-	3.49	26	−12	10
Left dorsolateral putamen	-	3.52	−24	0	10
Right precentral gyrus	8	4.32	42	4	34
Left precentral gyrus	6/4	3.87	−34	−8	42
Right supramarginal gyrus	7	4.37	40	−44	40
*Familial risk* *<* *no familial risk*					
Occipital pole	18	3.76	−2	−94	−16
*B. Task-related putamen connectivity*					
*Stimulant use* *<* *no stimulant use*					
Middle frontal gyrus	8	4.04	44	12	40
Superior frontal gyrus	6	3.33	−22	4	56
*Familial risk* *>* *no familial risk*					
Medial frontal cortex	10	3.52	2	50	−10
Frontal Pole	10	3.55	40	50	−8
Temporal pole	36	3.84	−20	4	−28
Brainstem	-	3.68	8	−24	−30
*Familial risk* *<* *no familial risk*					
Anterior cingulate cortex	24	3.45	4	−2	38
*Stimulant use X familial risk*					
Left precentral gyrus	4	4.38	−38	−14	48
Right precentral gyrus	6	3.64	30	−4	50
Postcentral gyrus	4/1	4.38	66	−6	30
Lateral occipital cortex	19	3.70	−18	−88	30
Lateral occipital cortex	19	3.59	26	−68	32

Regions are listed in order of cluster size from largest to smallest. (A) Summary of fMRI results for the monetary MID-condition. (B) PPI results showing the difference in functional connectivity with a seed in the bilateral putamen (MNI-coordinates: 26, −12, 10, and −24, 0, 10) during the reward anticipation

*BA* Broadmann Area

*This cluster extended to the paracingulate gyrus, anterior cingulate cortex (ACC), insula, pallidum, striatum, and thalamus

Whole brain neuroimaging analysis revealed significant main effects of both stimulant drug use and familial risk on task-related brain activation. We observed a main effect of *stimulants* in the bilateral precentral gyri, right supramarginal gyrus, and bilateral putamen. This effect was due to increased activation in these regions in stimulant drug users when compared with non-users (Fig. 3b). Notably, peak putamen clusters were located in the dorsolateral part of the putamen (MNI-coordinates x, y, z: 26, −12, 10 and −24, 0, 10)^56^. We also observed a main effect of *familial risk* in the left occipital pole, which was driven by greater task-related activation in participants without familial risk compared with participants with familial risk (Table 2). We observed no interaction between *stimulants* and *familial risk* in task-related brain activation.

Following the whole brain neuroimaging analysis, we subjected all significant clusters (Table 2) to an additional post-hoc analysis to address potentially confounding factors regarding drug-taking experiences and familial risk. The significant main effect of *stimulants* in the bilateral precentral gyri (right: F_1,158_ = 6.07, *p* = 0.015; left: F_1,158_ = 7.76, *p* = 0.006), right supramarginal gyrus (F_1,158_ = 6.35, *p* = 0.013), and bilateral putamen (right: F_1,158_ = 14.72, *p* < 0.001; left: F_1,158_ = 8.17, *p* = 0.005), and the main effect of *familial risk* in the left occipital pole (F_1,158_ = 8.32, *p* = 0.005), all survived this additional post-hoc analysis.

### Task-related brain functional connectivity

In view of the main effect of *stimulants* on task-related activation of the bilateral putamen and the purported role of the putamen in drug addiction^57^, we performed a PPI analysis to further explore the role of this region in reward anticipation. The GLM analysis, which used both *stimulants* and *familial risk* as the fixed factors, when conducted on PPI results, revealed an effect of *familial risk* on putamen functional connectivity strength to the right medial prefrontal cortex (mPFC), frontal pole, right ACC, left temporal lobe extending into the OFC, and brainstem. These results reflected increased putamen functional connectivity to the frontal pole, temporal pole, and brainstem, and decreased putamen functional connectivity to the ACC in individuals with familial risk compared with those without familial risk (Fig. 3c, Table 2). In addition to the main effect of *familial risk*, an effect of *stimulants* was observed in functional connectivity strength between the putamen and the right middle frontal gyrus/superior frontal gyrus, such that stimulant drug users showed decreased functional connectivity in this pathway (Table 2).

We also observed an interaction between *familial risk* and *stimulants* on putamen functional connectivity to the bilateral precentral gyrus, bilateral lateral occipital cortex, and right postcentral gyrus. The interaction effect was due to increased putamen functional connectivity in F−S+ participants and F+S− participants when compared with F+S+ and F–S– participants (Table 2).

Like the previous neuroimaging analysis, we subjected all significant clusters resulting from the PPI analysis to an additional post-hoc analysis to control for potentially confounding effects resulting from variations in stimulant drug-taking experiences and familial risk across the groups. These additional post-hoc analyses confirmed a significant main effect of *familial risk* in functional connectivity between the putamen and the right mPFC (F_1,158_ = 6.88, *p* = 0.010), frontal pole (F_1,158_ = 11.01, *p* = 0.001), right ACC (F_1,158_ = 7.31, *p* = 0.008), left temporal lobe extending to the OFC (F_1,158_ = 18.31, *p* < 0.001), and brainstem (F_1,158_ = 6.02, *p* = 0.015). However, the main effect of *stimulants*, encompassing the middle frontal gyrus (F_1,158_ = 2.54, *p* = 0.113) and superior frontal gyrus (F_1,158_ = 3.63, *p* = 0.059), did not reach significance.

The significant *familial risk*-by-*stimulant* interaction effects in functional connectivity between the putamen and the bilateral precentral gyrus (Right: F_1,158_ = 12.96, *p* < 0.001; Left: F_1,158_ = 12.07, *p* = 0.001), occipital cortex (Right: F_1,158_ = 16.77, *p* < 0.001; Left: F_1,158_ = 18.33, *p* = 0.005), and right postcentral gyrus (F_1,158_ = 14.38, *p* < 0.001) all survived additional post-hoc analyses.

No measures of task-related neural activation or functional connectivity were related to self-reported impulsivity, as measured by BIS-11 total score (*p* > 0.05; see Table S1).

## Discussion

The aim of the study was to disentangle the distinct and interacting effects of stimulant drug use and familial risk on reward processing. Risk associated with stimulant drug use encompasses both pre-existing factors related to the initiation of stimulant use and the effects of regular stimulant use. In contrast to previous studies, which investigated these effects separately, often using region of interest approaches, we examined these effects within a single model in the whole brain. Using this approach, we found that although task performance did not differ between groups, involvement of underlying corticostriatal circuitries indicated possible vulnerability and resilience factors related to stimulant drug addiction.

We found significantly increased task-related activation in the dorsolateral putamen, a region implicated in motor control^58^, rather than the ventral striatum, which is associated with reward processing and reinforcement. Regular use of stimulant drugs was significantly associated with increased task-related activation in motor circuitry. By contrast, familial risk was associated with altered corticostriatal functional connectivity between the putamen and brain regions implicated in reward processing, specifically the mPFC, OFC, and ACC. We also identified an interaction between familial risk and stimulant drug use in functional connectivity strength for the putamen and precentral and postcentral gyri, such that at-risk individuals (due to either familial risk or stimulant drug use) who did not develop addiction, showed increased functional connectivity strength within these regions. Together, our findings indicate that the two risk factors associated with stimulant drug use, i.e., familial and stimulant-related risk, are reflected by distinct functional brain changes during reward anticipation.

### Effects of stimulant drug use on reward processing

Although stimulant drug use was associated with differences in brain activation, it had no behaviorally measurable effects on MID-task performance. There are several approaches to interpreting neuroimaging findings not reflected in behavioral task performance^59^. Given that the MID-task was designed to preclude behavioral differences^34^, our findings may suggest underlying cognitive abnormalities that cannot be attributed to non-specific performance measures^60^. At a neural level, during an anticipatory period preceding a correct response, we identified in stimulant drug users compared with non-drug users, significantly increased task-related brain activation in the putamen and motor cortex (Fig. 3b)—brain regions implicated in motor response. Such activations in motor pathways during MID-task performance and cue-reactivity have been frequently reported in published work^25,31,61–66^, and are unsurprising given converging lines of evidence demonstrating the activation and alteration of motor-related brain regions by stimulant drugs^67^. Given that increased activation in motor pathways has been linked to stimulant drug-induced sensitization^24,68^ and underlying progressive recruitment of sensorimotor and dorsal striatal regions^69,70^ in stimulant drug users (an effect shown to persist for several months following stimulant drug exposure^69^), it is tempting to speculate that increased activation seen in stimulant-using individuals in the current study might reflect enhanced ventral-to-dorsal progression of striatal recruitment facilitated by stimulant drugs^71,72^.

In light of previous reports on striatal function during the MID-task, we predicted blunted ventral striatal activation in stimulant drug users^25,49^. However, unlike the robust changes in motor circuitry, we found no measurable effects of stimulant drug use on task-related activation patterns within the ventral striatum–widely considered to be a neural correlate for reward anticipation^34^–even though this structure was strongly activated during the MID-task performance in all participants. It is noteworthy that task-related differences in ventral striatal activation following stimulant abuse have not been consistently reported^25,61,62,73–77^, which may be due to variations in sample characteristics and experimental design^78^ such as the recency of drug use^79^. Accordingly, although blunted activation in the ventral striatum is frequently reported in abstinent stimulant-dependent individuals^63,80–82^, such differences are often not observed^76,77,83,84^ in active drug users, consistent with present findings.

### Effects of familial risk on reward processing

Similar to the effects of stimulant drug use, familial risk had no measurable effect on MID-task performance. However, unlike stimulant drug use, familial risk also had no measurable impact on task-related brain activation in reward-related brain regions. This observation is consistent with previous fMRI studies in individuals with familial risk^85–87^. Our data suggest that familial risk is conveyed in task-related corticostriatal functional connectivity (Fig. 3c). Specifically, we observed altered functional connectivity between the putamen and the ACC, OFC, and mPFC–brain regions associated with the processing of monetary rewards^34,35^–in participants with familial risk, irrespective of whether they used stimulant drugs.

These findings are interesting given a bulk of research demonstrating functional and structural pathways between the ventral putamen and frontal regions such as the mPFC, OFC, and ACC^88,89^. Accordingly, the ventral putamen is involved in a frontal network that is recruited during response monitoring or goal-directed control required for the processing of reward^90^. These observations strongly suggest that the putamen, in addition to being a motor structure, is involved in higher-level cognitive functions^56^. In particular, the OFC, mPFC, and ACC have been implicated in an inhibition network consisting of cortical motor regions and basal ganglia structures, including the putamen^91,92^. It is possible that aberrant functioning of these corticostriatal networks confers vulnerability on individuals with family risk, altering higher-cognitive functioning and increasing their susceptibility to the development of addiction.

Abnormal task-related corticostriatal functional connectivity demonstrated here, and elsewhere, in both addicted^93^ and vulnerable adolescents^86^, adds to the body of evidence supporting endophenotypes in striatal and prefrontal cortical brain regions. Prior findings of enlarged putamen^12^ and altered prefrontal cortical white matter in sibling pairs^43^, along with the present findings, suggest that such endophenotypes may consist of both structural and functional striatal abnormalities. Taken together with the effects of stimulant drug use, our results suggest that stimulant drug addiction may impact the function of structures contained within brain pathways already compromised by familial risk, thereby exacerbating pre-existing functional deficits, perhaps leading to the development of addiction.

### Interaction between stimulant drug use and familial risk during reward processing

In the present study, we observed an interaction on task-related functional connectivity between the putamen and the precentral and postcentral gyri in non-addicted stimulant drug users and non-stimulant drug users with familial risk. This interaction was characterized by an increase in functional connectivity strength to the precentral and postcentral gyri, which are involved in motor and somatosensory functions, possibly indicating compensatory or resiliency mechanisms. Potential resilience, in the form of low sensation-seeking traits, has been previously reported in unaffected siblings^44,48^, which may reflect lack of motivation to initiate drug use. In previous studies, individuals with familial risk demonstrated increased striatal functional connectivity strength to attention- and motor-related structures, including the precentral and postcentral gyri, during reward anticipation^86^. However, such studies were unable to determine whether such changes represented addiction risk or resilience. Here we provide additional evidence that functional connectivity strength between striatal regions and motor-related structures when anticipating a reward may represent a mechanism for resilience in individuals with either familial risk or risks associated with the use of stimulants. Such changes in motor-related structures may confer increased motor control, allowing these resilient individuals to cope with/resist the addictive properties of drug use or mitigate pre-existing impulsive tendencies^94^.

### Limitations and implications

To the best of our knowledge, the present results demonstrate for the first time distinct effects of stimulant drug use and familial risk on task-related putamen activation and functional connectivity of the putamen, respectively. Our study benefitted from a rigorous statistical approach and was conducted in a large sample with relatively stringent statistical thresholds. However, we also acknowledge several limitations of this study. Potentially confounding influences in stimulant drug-taking experiences and familial risk in the selected statistical model were addressed using additional post-hoc analyses, which are not always ideal for controlling for differences in incidental variables as such differences may indicate differences in other relevant variables that were not assessed. We also acknowledge that stimulant drug users were separated based on their familial risk, yet familial risk is only one, albeit important, risk factor for the development of addiction. Other possible risk factors may encompass neurobiological or socioeconomic differences that may exist between dependent and non-dependent stimulant drug users^45,46,95,96^, rendering some individuals more vulnerable to developing addiction than others. Furthermore, the design of our study does not allow for the identification of the specific aspects of familial or drug use risks responsible for the current findings.

Reward-related striatal function has been associated with measures of addiction recovery and rehabilitation^97^. In the absence of medically proven pharmacological treatments, therapeutic interventions for stimulant drug addiction currently rely on psychosocial and family-based approaches^98^, which focus on previously identified familial environmental risk-factors^28^ but do not take into account associated neurobiological risk-factors. Embedding the emerging knowledge of neurobiological vulnerability factors that are shared between family members into family-based interventions may increase therapeutic efficacy, perhaps by facilitating the delivery of prevention and intervention programs during childhood and adolescence. Of particular relevance, we identified altered corticostriatal pathways as a candidate endophenotype for stimulant drug addiction. To fully realize the implications of this result, future research should attempt to characterize the behaviors supported by these pathways, and how they contribute to the development of addiction. Thus, the identification of endophenotypes, as well as resiliency mechanisms, will contribute to the synthesis of intervention strategies and the development of targeted therapies by providing distinct neural biomarkers that may help predict treatment response or successful/unsuccessful abstinence in addiction.

## Supplementary information

Supplemental material

## References

[1] Leshner AI (1997). Addiction is a brain disease, and it matters. Science.

[2] Nestler EJ (2005). Is there a common molecular pathway for addiction?. Nat. Neurosci..

[3] Haber SN, Knutson B (2010). The reward circuit: linking primate anatomy and human imaging. Neuropsychopharmacol. Publ. Am. Coll. Neuropsychopharmacol..

[4] Taylor SB, Lewis CR, Olive MF (2013). The neurocircuitry of illicit psychostimulant addiction: acute and chronic effects in humans. Subst. Abus. Rehabil..

[5] Volkow ND, Fowler JS, Wang GJ, Swanson JM (2004). Dopamine in drug abuse and addiction: results from imaging studies and treatment implications. Mol. Psychiatry.

[6] Berridge KC (2007). The debate over dopamine’s role in reward: the case for incentive salience. Psychopharmacol. (Berl.).

[7] Berridge KC, Robinson TE (1995). The mind of an addicted brain: neural sensitization of wanting versus liking. Curr. Dir. Psychol. Sci..

[8] Merikangas KR (1998). Familial transmission of substance use disorders. Arch. Gen. Psychiatry.

[9] Bevilacqua L, Goldman D (2009). Genes and addictions. Clin. Pharmacol. Ther..

[10] Hart AB, de Wit H, Palmer AA (2012). Genetic factors modulating the response to stimulant drugs in humans. Curr. Top. Behav. Neurosci..

[11] Ersche KD (2012). Cognitive dysfunction and anxious-impulsive personality traits are endophenotypes for drug dependence. Am. J. Psychiatry.

[12] Ersche KD (2012). Abnormal brain structure implicated in stimulant drug addiction. Science.

[13] Volkow ND (2006). High levels of dopamine D2 receptors in unaffected members of alcoholic families: possible protective factors. Arch. Gen. Psychiatry.

[14] Casey KF (2014). Reduced dopamine response to amphetamine in subjects at ultra-high risk for addiction. Biol. Psychiatry.

[15] Trifilieff P, Martinez D (2014). Blunted dopamine release as a biomarker for vulnerability for substance use disorders. Biol. Psychiatry.

[16] Andrews MM (2011). Individuals family history positive for alcoholism show functional magnetic resonance imaging differences in reward sensitivity that are related to impulsivity factors. Biol. Psychiatry.

[17] Crane NA (2017). Preliminary evidence for disrupted nucleus accumbens reactivity and connectivity to reward in binge drinkers. Alcohol Alcohol Oxf. Oxfs..

[18] Nees F (2012). Determinants of early alcohol use in healthy adolescents: the differential contribution of neuroimaging and psychological factors. Neuropsychopharmacol. Publ. Am. Coll. Neuropsychopharmacol..

[19] Heinrich A (2016). Prediction of alcohol drinking in adolescents: Personality-traits, behavior, brain responses, and genetic variations in the context of reward sensitivity. Biol. Psychol..

[20] Whelan R (2014). Neuropsychosocial profiles of current and future adolescent alcohol misusers. Nature.

[21] White TL (2017). Beyond sensation seeking: a conceptual framework for individual differences in psychostimulant drug effects in healthy humans. Curr. Opin. Behav. Sci..

[22] Arens CR, White TL, Massengill N (2014). Attitudinal factors protective against youth smoking: an integrative review. J. Nurs. Scholarsh. Publ. Sigma Theta Tau Int Honor Soc. Nurs..

[23] Robert GH, Schumann G (2017). Reinforcement related behaviors and adolescent alcohol abuse: from localized brain structures to coordinated networks. Curr. Opin. Behav. Sci..

[24] Leyton M (2007). Conditioned and sensitized responses to stimulant drugs in humans. Prog. Neuropsychopharmacol. Biol. Psychiatry.

[25] Luijten M, Schellekens AF, Kühn S, Machielse MWJ, Sescousse G (2017). Disruption of reward processing in addiction: an image-based meta-analysis of functional magnetic resonance imaging studies. JAMA Psychiatry.

[26] Gottesman II, Gould TD (2003). The endophenotype concept in psychiatry: etymology and strategic intentions. Am. J. Psychiatry.

[27] Galea S, Vlahov D (2002). Social determinants and the health of drug users: socioeconomic status, homelessness, and incarceration. Public Health Rep. Wash. DC1974.

[28] Zimić JI, Jukić V (2012). Familial risk factors favoring drug addiction onset. J. Psychoact. Drugs.

[29] Toomey R (2003). A twin study of the neuropsychological consequences of stimulant abuse. Arch. Gen. Psychiatry.

[30] Keyes MA (2009). The enrichment study of the Minnesota twin family study: increasing the yield of twin families at high risk for externalizing psychopathology. Twin Res Hum. Genet J. Int Soc. Twin Stud..

[31] Volkow ND (2006). Cocaine cues and dopamine in dorsal striatum: mechanism of craving in cocaine addiction. J. Neurosci. J. Soc. Neurosci..

[32] Volkow ND, Fowler JS, Wang GJ, Swanson JM, Telang F (2007). Dopamine in drug abuse and addiction: results of imaging studies and treatment implications. Arch. Neurol..

[33] Goldman D, Oroszi G, Ducci F (2005). The genetics of addictions: uncovering the genes. Nat. Rev. Genet..

[34] Knutson B, Fong GW, Adams CM, Varner JL, Hommer D (2001). Dissociation of reward anticipation and outcome with event-related fMRI. Neuroreport.

[35] Knutson B, Greer SM (2008). Anticipatory affect: neural correlates and consequences for choice. Philos. Trans. R. Soc. Lond. B. Biol. Sci..

[36] First M., Spitzer R., Williams J. Structured Clinical Interview for DSM-IV-TR Axis I Disorders, Research Version, Non-Patient Research (SCID-I/NP). 2002.

[37] American Psychiaatric Association. Diagnostic and Statistical Manual of Mental Disorders. 2002.

[38] Bierut LJ, Strickland JR, Thompson JR, Afful SE, Cottler LB (2008). Drug use and dependence in cocaine dependent subjects, community-based individuals, and their siblings. Drug Alcohol. Depend..

[39] Brown J, Cohen P, Johnson JG, Salzinger S (1998). A longitudinal analysis of risk factors for child maltreatment: findings of a 17-year prospective study of officially recorded and self-reported child abuse and neglect. Child Abus. Negl..

[40] Walsh C, MacMillan HL, Jamieson E (2003). The relationship between parental substance abuse and child maltreatment: findings from the Ontario Health Supplement. Child Abus. Negl..

[41] Bernstein DP, Ahluvalia T, Pogge D, Handelsman L (1997). Validity of the Childhood Trauma Questionnaire in an adolescent psychiatric population. J. Am. Acad. Child Adolesc. Psychiatry.

[42] Preston KL (2002). Urinary elimination of cocaine metabolites in chronic cocaine users during cessation. J. Anal. Toxicol..

[43] Morein-Zamir S, Simon Jones P, Bullmore ET, Robbins TW, Ersche KD (2013). Prefrontal hypoactivity associated with impaired inhibition in stimulant-dependent individuals but evidence for hyperactivation in their unaffected siblings. Neuropsychopharmacol. Publ. Am. Coll. Neuropsychopharmacol..

[44] Ersche KD (2013). Distinctive personality traits and neural correlates associated with stimulant drug use versus familial risk of stimulant dependence. Biol. Psychiatry.

[45] Smith DG, Simon Jones P, Bullmore ET, Robbins TW, Ersche KD (2014). Enhanced orbitofrontal cortex function and lack of attentional bias to cocaine cues in recreational stimulant users. Biol. Psychiatry.

[46] Morein-Zamir S, Simon Jones P, Bullmore ET, Robbins TW, Ersche KD (2015). Take it or leave it: prefrontal control in recreational cocaine users. Transl. Psychiatry.

[47] Smith DG, Jones PS, Bullmore ET, Robbins TW, Ersche KD (2013). Cognitive control dysfunction and abnormal frontal cortex activation in stimulant drug users and their biological siblings. Transl. Psychiatry.

[48] Ersche KD, Turton AJ, Pradhan S, Bullmore ET, Robbins TW (2010). Drug addiction endophenotypes: impulsive versus sensation-seeking personality traits. Biol. Psychiatry.

[49] Knutson B, Westdorp A, Kaiser E, Hommer D (2000). FMRI visualization of brain activity during a monetary incentive delay task. Neuroimage.

[50] Sacchet MD, Knutson B (2013). Spatial smoothing systematically biases the localization of reward-related brain activity. Neuroimage.

[51] Rose RJ, Dick DM, Viken RJ, Kaprio J (2001). Gene-environment interaction in patterns of adolescent drinking: regional residency moderates longitudinal influences on alcohol use. Alcohol. Clin. Exp. Res..

[52] Tobler PN, Fletcher PC, Bullmore ET, Schultz W (2007). Learning-related human brain activations reflecting individual finances. Neuron.

[53] Konova AB (2016). Converging effects of cocaine addiction and sex on neural responses to monetary rewards. Psychiatry Res..

[54] Miller GA, Chapman JP (2001). Misunderstanding analysis of covariance. J. Abnorm. Psychol..

[55] Eklund A, Nichols TE, Knutsson H (2016). Cluster failure: Why fMRI inferences for spatial extent have inflated false-positive rates. Proc. Natl Acad. Sci. USA.

[56] Postuma RB, Dagher A (2006). Basal ganglia functional connectivity based on a meta-analysis of 126 positron emission tomography and functional magnetic resonance imaging publications. Cereb. Cortex N. Y N. 1991.

[57] Everitt BJ, Robbins TW (2013). From the ventral to the dorsal striatum: devolving views of their roles in drug addiction. Neurosci. Biobehav. Rev..

[58] Alexander GE, DeLong MR, Strick PL (1986). Parallel organization of functionally segregated circuits linking basal ganglia and cortex. Annu. Rev. Neurosci..

[59] Fletcher PC (2004). Functional neuroimaging of psychiatric disorders: exploring hidden behaviour. Psychol. Med..

[60] Wilkinson D, Halligan P (2004). The relevance of behavioural measures for functional-imaging studies of cognition. Nat. Rev. Neurosci..

[61] Konova AB (2012). Structural and behavioral correlates of abnormal encoding of money value in the sensorimotor striatum in cocaine addiction. Eur. J. Neurosci..

[62] Jager G, Block RI, Luijten M, Ramsey NF (2013). Tentative evidence for striatal hyperactivity in adolescent cannabis-using boys: a cross-sectional multicenter fMRI study. J. Psychoact. Drugs.

[63] Schouw MLJ (2013). Dopaminergic dysfunction in abstinent dexamphetamine users: results from a pharmacological fMRI study using a reward anticipation task and a methylphenidate challenge. Drug Alcohol. Depend..

[64] Nestor L, Hester R, Garavan H (2010). Increased ventral striatal BOLD activity during non-drug reward anticipation in cannabis users. Neuroimage.

[65] van Holst RJ, Clark L, Veltman DJ, van den Brink W, Goudriaan AE (2014). Enhanced striatal responses during expectancy coding in alcohol dependence. Drug Alcohol. Depend..

[66] Rose EJ (2013). Acute nicotine differentially impacts anticipatory valence- and magnitude-related striatal activity. Biol. Psychiatry.

[67] Stewart J, Badiani A (1993). Tolerance and sensitization to the behavioral effects of drugs. Behav. Pharmacol..

[68] Vanderschuren LJ, Kalivas PW (2000). Alterations in dopaminergic and glutamatergic transmission in the induction and expression of behavioral sensitization: a critical review of preclinical studies. Psychopharmacol. (Berl.).

[69] Boileau I (2006). Modeling sensitization to stimulants in humans: an [11C]raclopride/positron emission tomography study in healthy men. Arch. Gen. Psychiatry.

[70] Ito R, Dalley JW, Robbins TW, Everitt BJ (2002). Dopamine release in the dorsal striatum during cocaine-seeking behavior under the control of a drug-associated cue. J. Neurosci. J. Soc. Neurosci..

[71] Porrino LJ, Daunais JB, Smith HR, Nader MA (2004). The expanding effects of cocaine: studies in a nonhuman primate model of cocaine self-administration. Neurosci. Biobehav. Rev..

[72] Everitt BJ, Robbins TW (2005). Neural systems of reinforcement for drug addiction: from actions to habits to compulsion. Nat. Neurosci..

[73] Bjork JM, Smith AR, Hommer DW (2008). Striatal sensitivity to reward deliveries and omissions in substance dependent patients. Neuroimage.

[74] Bjork JM, Smith AR, Chen G, Hommer DW (2012). Mesolimbic recruitment by nondrug rewards in detoxified alcoholics: effort anticipation, reward anticipation, and reward delivery. Hum. Brain. Mapp..

[75] Jia Z (2011). An initial study of neural responses to monetary incentives as related to treatment outcome in cocaine dependence. Biol. Psychiatry.

[76] Jansma JM (2013). THC reduces the anticipatory nucleus accumbens response to reward in subjects with a nicotine addiction. Transl. Psychiatry.

[77] Patel KT (2013). Robust changes in reward circuitry during reward loss in current and former cocaine users during performance of a monetary incentive delay task. Biol. Psychiatry.

[78] Balodis IM, Potenza MN (2015). Anticipatory reward processing in addicted populations: a focus on the monetary incentive delay task. Biol. Psychiatry.

[79] Balodis IM (2016). Neurofunctional reward processing changes in cocaine dependence during recovery. Neuropsychopharmacol. Publ. Am. Coll. Neuropsychopharmacol..

[80] Beck A (2009). Ventral striatal activation during reward anticipation correlates with impulsivity in alcoholics. Biol. Psychiatry.

[81] Forbes EE, Rodriguez EE, Musselman S, Narendran R (2014). Prefrontal response and frontostriatal functional connectivity to monetary reward in abstinent alcohol-dependent young adults. PLoS ONE.

[82] van Hell HH (2010). Chronic effects of cannabis use on the human reward system: an fMRI study. Eur. Neuropsychopharmacol. J. Eur. Coll. Neuropsychopharmacol..

[83] Enzi B (2015). Alterations of monetary reward and punishment processing in chronic cannabis users: an FMRI study. PLoS ONE.

[84] Filbey FM, Dunlop J, Myers US (2013). Neural effects of positive and negative incentives during marijuana withdrawal. PLoS ONE.

[85] Bjork JM, Knutson B, Hommer DW (2008). Incentive-elicited striatal activation in adolescent children of alcoholics. Addict. Abingdon Engl..

[86] Weiland BJ (2013). Accumbens functional connectivity during reward mediates sensation-seeking and alcohol use in high-risk youth. Drug Alcohol. Depend..

[87] Müller KU (2015). No differences in ventral striatum responsivity between adolescents with a positive family history of alcoholism and controls. Addict. Biol..

[88] Di Martino A (2008). Functional connectivity of human striatum: a resting state FMRI study. Cereb. Cortex N. Y N. 1991.

[89] Lehéricy S (2004). Diffusion tensor fiber tracking shows distinct corticostriatal circuits in humans. Ann. Neurol..

[90] Haber SN (2016). Corticostriatal circuitry. Dialog-. Clin. Neurosci..

[91] Everitt BJ, Robbins TW (2016). Drug addiction: updating actions to habits to compulsions ten years on. Annu. Rev. Psychol..

[92] Hampshire A, Sharp DJ (2015). Contrasting network and modular perspectives on inhibitory control. Trends Cogn. Sci..

[93] Jollans L (2016). Ventral striatum connectivity during reward anticipation in adolescent smokers. Dev. Neuropsychol..

[94] Mechelmans DJ (2017). Reward sensitivity and waiting impulsivity: shift towards reward valuation away from action control. Int. J. Neuropsychopharmacol..

[95] Hulka LM (2014). Altered social and non-social decision-making in recreational and dependent cocaine users. Psychol. Med..

[96] Quednow BB (2017). Stable self-serving personality traits in recreational and dependent cocaine users. PLoS ONE.

[97] Yip SW (2014). Pretreatment measures of brain structure and reward-processing brain function in cannabis dependence: an exploratory study of relationships with abstinence during behavioral treatment. Drug Alcohol. Depend..

[98] European Monitoring Centre for Drug Addiction. Treatment for Cocaine Dependence: Reviewing Current Evidence. 2014.

