# Supplementary material for: Effects of familial risk and stimulant drug use on the anticipation of monetary reward: an fMRI study
Source: Transl Psychiatry. 2019 Feb 4;9:65. doi: 10.1038/s41398-019-0399-4 (PMC6362203; doi:10.1038/s41398-019-0399-4)
Supplement: Supplementary file 1 — Supplemental material [file 41398_2019_399_MOESM1_ESM.docx]

**Effects of familial risk and stimulant drug use on the anticipation of monetary reward: an fMRI study**

Alanna L. Just, MPhil; Chun Meng, PhD; Dana G. Smith, PhD; Edward T. Bullmore, PhD;
Trevor W. Robbins, PhD; Karen D. Ersche, PhD

***Supplementary Material***

**SUPPLEMENTARY METHODS AND MATERIALS**

**Study Sample**

F+S+ participants last used stimulant drugs an average of 2.2 (±2.4 SD) days prior to testing. Several F+S+ participants were prescribed medication (painkillers N=1; benzodiazepines N=3; antidepressants N=10; Dexedrine® N=3, mean dose= 38.3mg ± 24.66 SD) and methadone substitution (N=19, mean does= 53.4mg ± 24.61 SD), and two siblings were prescribed antidepressants. All stimulant using controls reported the use of powdered cocaine exclusively in social settings. Control participants reported no use of any medications.

**Neuroimaging Data Acquisition**

The neuroimaging data were acquired at the Wolfson Brain Imaging Centre, University of Cambridge, in one run on a Siemens TIM Trio 3-Tesla scanner (Siemens Medical Solutions, Erlangen, Germany). Whole-brain echo planer images (EPIs) with blood oxygen level-dependent contrast were collected using the following parameters: 2000ms repetition time (TR); 30ms echo time (TE); 78⁰ flip angle; 32 slices each 3mm thick, 0.75mm inter-slice gap; 64x64 matrix; 192x192mm field of view (FOV) yielding 3x3mm in-plane resolution, and the number of volumes ranged from 278 to 305. T1-weighted scans were acquired for registration purposes (176 slices of 1mm thickness, TR=2300ms; TE=2.98ms, TI=900ms, flip angle=9⁰, FOV=240x256mm).

**Neuroimaging Data Analysis**

Our neuroimaging analyses consisted of two main stages: 1) task activation analysis to identify brain regions involved in reward processing and effects of interest (i.e. main effects of familial risk, stimulant drug use, and their interaction); 2) psychophysiological interaction (PPI) analysis to further explore striatal functional connectivity related to reward anticipation and effects of interest.

Imaging data were analysed in FMRIB's Software Library (FSL, v-5.0.9, https://fsl.fmrib.ox.ac.uk/fsl). Pre-processing and statistical analyses were conducted by using FMRI Expert Analysis Tool (FEAT, version 6.0). The first five images of each participant’s functional scan were discarded for the stabilization of longitudinal magnetization. The remaining images were pre-processed using the following steps: motion correction, skull stripping, spatial smoothing with a Gaussian kernel (FWHM = 5 mm), grand-mean scaling, and high-pass temporal filtering with a 100s cut-off. After pre-processing, visual inspection and quantitative assessment were used for quality control. Summarized metrics for each participant’s data included tSNR (temporal signal-to-noise^1^) and DVARS (using FSL’s fsl_motion_outlier tool^2^. Severe artefacts were found in five participants (3 F+S+, 1 F+S-, 1 F-S-; DVARS > 45, or tSNR < 20) who were, as mentioned above, excluded from further analysis. For each participant, a non-linear warp-field for normalizing functional image, via T1 anatomical image, to standard MNI152 space was calculated by combining boundary-based registration (FSL’s FLIRT) and nonlinear registration (FSL’s FNIRT).

Statistical analyses were conducted at the first and second levels by using FMRI Expert Analysis Tool (FEAT). At the first level, a general linear model (GLM) analysis was conducted for each participant, including regressors for the stimulus (monetary reward, drug reward, and respective neutral rewards), and by type (anticipation, successful feedback, and unsuccessful feedback). In total, 12 explanatory variables (EVs) were constructed and then convolved with a double-gamma hemodynamic response function, to be included in the first-level GLM. A temporal derivative was also added for each EV. Additional 24 motion-related EVs (including six motion estimates and their derivative and quadratic forms) were added to account for covariate effects of no interest. The first-level analyses provided one statistical map per contrast per participant.

At the second level, 160 participants’ statistical maps were warped into standard MNI152 space with 2x2x2mm voxel by using T1-based normalization procedure and then entered into group analyses by using FSL-FLAME for mixed effects modelling. First, the main effect of each contrast collapsing across groups was computed using one-sample t-tests, in order to reveal task activation patterns. Second, four groups were modelled in the ANCOVA model to assess main effects of *stimulants and familial risk* as well as their interaction for each contrast.

To further explore functional connectivity during reward anticipation, post-hoc exploratory PPI analysis was used between striatal seed regions of interests (ROI) and whole brain. The seed ROI was defined by the peak coordinates of significant striatal clusters with a 4mm radius. For each participant, the average time course of the seed ROI, and its product with the HRF-convolved regressor of selected task contrast (i.e. PPI regressor), were added into the first-level GLM. The first-level statistical maps of PPI regressor were calculated and subsequently entered into second-level ANCOVA analysis.

In light of recent concern about inflated false positives in cluster inference^3,4^, we reported our main imaging findings based on whole-brain group analyses and at a relatively stringent threshold. The initial cluster forming threshold was set at Z>2.58, i.e. p<0.005. Cluster-level corrected p<0.00015 was set as the significance level by using FSL’s easythresh function. Following the identification of significant clusters, the mean contrast estimates within clusters were extracted from each participant, and then imported into SPSS for post-hoc correlation analyses.

**SUPPLEMENTARY RESULTS**

**Demographics**

We found significant group differences in nicotine, cannabis and alcohol use, which were controlled for using an additional post-hoc test (see Methods). Specifically, we found an effect of *familial risk* on nicotine consumption (F_1,84_=4.01, p=0.049), indicating greater nicotine consumption in the sibling pairs. Post-hoc analysis revealed main effects of *familial risk* (F_1,155_=9.36, p=0.003) and *stimulants* (F_1,155_=22.65, p<0.001), and a *stimulants*-by-*familial risk* interaction (F_1,155_=5.91, p=0.016) on alcohol consumption (AUDIT scores). This interaction effect was driven by greater alcohol consumption in F+S+ participants, as is common with drug-dependent populations^5^.

**Neuroimaging data**

Effects of familial risk and stimulants: reward feedback

We observed an interaction between *familial risk* and *stimulants* during feedback of successful trials on right postcentral gyrus (p<0.0001, MNI coordinates: 46, -16, 34) and left precentral gyrus (p<0.0001, MNI coordinates: -52, -6, 34) activation such that familial stimulant drug users and non-familial stimulant drug users displayed increased or decreased activation, respectively, compared with all non-using participants. There was also an interaction between *familial risk* and *stimulants* during feedback of unsuccessful trials on right anterior cingulate cortex activation (p<0.0001, xyz-MNI coordinates: 4, 8, 38). There were no significant main effects of *stimulants* or *familial risk* during successful or unsuccessful feedback.

ROI analysis

In addition to the whole brain analysis, we also conducted a separate analysis on brain activation during money anticipation within a striatum mask. Collapsed across all groups, we observed peak clusters in the left ventral striatum (MNI coordinates: -8, 10, -6; FWE corrected p = 0.006) and the right ventral striatum (MNI coordinates: 8, 6, -2; FWE corrected p = 0.007) in response to money anticipation. These confirmed the results reported in the whole brain analyses.

Using the striatum mask, we found significant main effect of *stimulants* in the dorsolateral part of the bilateral putamen (xyz-MNI coordinates: 26, -12, 10 and -24, 0, 10) identical to that observed in the whole brain analysis. There were no significant main effects of *familiality* or interaction effects using the striatum mask.

We also used a smaller ventral striatum mask centered on the nucleus accumbens (Harvard-Oxford Atlas). ANOVA run using this mask yielded no significant results even when a more liberal threshold (uncorrected p < 0.005, Z > 2.58) was used.

**Correlational Analysis**

We examined possible correlations between impulsivity and task-related neural activation and functional connectivity using the Barratt Impulsiveness Scale (BIS-11). All tests were two tailed and a p-value < 0.05 was considered significant. No measures of task-related neural activation or functional connectivity were related to impulsivity (p > 0.05; see supplementary Table 1).

**Table S1:** Relationships between trait-impulsivity (BIS-11 total scores)
and task-related brain activation.

| **Brain region** | **r-value** | **p-value** |
| --- | --- | --- |
|  |  |  |
| **A. Task-related activation** |  |  |
|  |  |  |
| *Stimulant use > no stimulant use* |  |  |
| Right dorsolateral putamen | 0.172 | 0.110 |
| Left dorsolateral putamen | 0.093 | 0.393 |
| Right precentral gyrus | 0.028 | 0.800 |
| Left precentral gyrus | 0.055 | 0.615 |
| Right supramarginal gyrus | -0.054 | 0.618 |
|  |  |  |
| *Familial risk < no familial risk* |  |  |
| Occipital pole | 0.092 | 0.399 |
|  |  |  |
| **B. Task-related putamen connectivity** | | |
|  |  |  |
| *Stimulant use < no stimulant use* |  |  |
| Middle frontal gyrus | -0.053 | 0.628 |
| Superior frontal gyrus | -0.085 | 0.435 |
|  |  |  |
| *Familial risk > no familial risk* |  |  |
| Medial frontal cortex | -0.072 | 0.510 |
| Frontal Pole | -0.095 | 0.381 |
| Temporal pole | -0.037 | 0.733 |
| Brainstem | -0.140 | 0.196 |
|  |  |  |
| *Familial risk < no familial risk* |  |  |
| Anterior cingulate cortex | -0.047 | 0.633 |
|  |  |  |
| *Stimulant use X familial risk* |  |  |
| Left precentral gyrus | -0.054 | 0.620 |
| Right precentral gyrus | 0.022 | 0.837 |
| Postcentral gyrus | -0.139 | 0.199 |
| Lateral occipital cortex | -0.005 | 0.966 |
| Lateral occipital cortex | -0.103 | 0.344 |
|  |  |  |

**SUPPLEMENTARY REFERENCES**

1. Murphy K, Bodurka J, Bandettini PA. How long to scan? The relationship between fMRI temporal signal to noise ratio and necessary scan duration. *NeuroImage* 2007; **34**: 565–74.
2. Power JD, Barnes KA, Snyder AZ, Schlaggar BL, Petersen SE. Spurious but systematic correlations in functional connectivity MRI networks arise from subject motion. *NeuroImage* 2012; **59**: 2142–54.
3. Eklund A, Nichols TE, Knutsson H. Cluster failure: Why fMRI inferences for spatial extent have inflated false-positive rates. *Proceedings of the National Academy of Sciences* 2016; **113**: 7900–7905.
4. Roiser JP, Linden DE, Gorno-Tempini ML, Moran RJ, Dickerson BC, Grafton ST. Minimum statistical standards for submissions to Neuroimage: Clinical. *NeuroImage: Clinical* 2016; **12**: 1045–1047.
5. Moss HB, Goldstein RB, Chen CM, Yi H-Y. Patterns of use of other drugs among those with alcohol dependence: Associations with drinking behavior and psychopathology. *Addict Behav* 2015; **50**: 192–198.
